# Change-of-Direction Performance in Elite Soccer Players: Preliminary Analysis According to Their Playing Positions

**DOI:** 10.3390/ijerph17228360

**Published:** 2020-11-12

**Authors:** Dorsaf Sariati, Raouf Hammami, Mokhtar Chtara, Alessandro Zagatto, Daniel Boullosa, Cain C. T. Clark, Anthony C. Hackney, Urs Granacher, Nizar Souissi, Hassane Zouhal

**Affiliations:** 1 Higher Institute of Sport and Physical Education of Ksar-Said, University of La Manouba, Manouba 2010, Tunisia; dor80sar18@yahoo.com (D.S.); raouf.cnmss@gmail.com (R.H.); niz.souissi@gmail.com (N.S.); 2 Research Laboratory, Education, Motricity, Sports and Health, University of Sfax, Sfax 3000, Tunisia; 3 Tunisian Research Laboratory, Sport Performance Optimization, National Center of Medicine and Science in Sports (CNMSS), Tunis 1003, Tunisia; chtaramoktar@yahoo.fr; 4 Faculty of Sciences, Department of Physical Education, UNESP-São Paulo State University, Bauru 01049-010, Brazil; azagatto@yahoo.com.br; 5 INISA, Federal University of Mato Grosso Do Sul, Campo Grande 79070-900, Brazil; d_boullosa@yahoo.es; 6 Centre for Intelligent Healthcare, Coventry University, Coventry CV1 5FB, UK; Cain.Clark@coventry.ac.uk; 7 Department of Exercise & Sport Science, University of North Carolina, Chapel Hill, NC 27599, USA; thackney@med.unc.edu; 8 Division of Training and Movement Sciences, University of Potsdam, 14469 Potsdam, Germany; 9 M2S (Laboratoire Mouvement, Sport, Santé)—EA 1274, University of Rennes, F-35000 Rennes, France

**Keywords:** CoD, players’ position, football, dynamic performances

## Abstract

Our objective was to examine the relationship between change of direction (CoD) performance, with (CoDb), and without the ball (CoDwb), and selected measures of physical fitness (jump performance, speed, balance) in elite soccer players, according to players’ positions. Forty elite male soccer players performed the change-of-direction and acceleration test (CODAT) with (CODATb), and without the ball (CODATwb), 5- and 20-m sprint tests, the 5-jump test (5JT), and the Y-balance test (YBT). Analyses of the whole sample showed significant correlations between all CODAT measures (CODATwb and CODATb, respectively) and sprint 5-m (r = 0.72, *p* < 0.001; r = 0.52, *p* < 0.01), sprint 20-m (r = 0.54, *p* < 0.03; r = 0.45, *p* < 0.05), jump (r = −0.62, *p* < 0.01; r = −0.64, *p* < 0.01) and balance (r = −0.50, *p* < 0.01; r = −0.83, *p* < 0.001) performances. Correlations were significantly different between player positions (defender, midfielder and striker). When examining the entire sample, the single best predictor of CODATwb was performance in the 5-m test with an explained variance of 52% (*p* < 0.001). For CODATb, the Y-balance performance explained 68% of the variance of performance (*p* < 0.001). In conclusion, soccer coaches and fitness trainers are advised to improve players’ CoD using neuromuscular training that mimic crucial match actions. Meanwhile, CoD testing and training should be designed in line with the demands of playing position.

## 1. Introduction

Soccer is a multifaceted team sport that requires well-developed speed, agility, balance and power to be played at an elite level [1,2,3,4]. In addition to possessing high levels of physical fitness, knowledge about the game and a good decision-making process are the key abilities [2,5]. The modern game is characterized by dynamic movements such as short and long sprints, impulsive reactions and quick changes-of-direction (CoD) [6]. Actions such as these are known to determine soccer performance and can be divided into categories related to sub-maximal speed, acceleration and CoD [7]. High level CoD performance requires a combination of speed, balance, muscular power and coordination [8].

Previous cross-sectional studies examined the relationship between jump [9,10], balance [9,11,12], sprint [7,11] and CoD performances in soccer. According to these studies, jump performance accounted for 45% of the variance in CoD performance in elite soccer players [13]. In addition, there is evidence that sprint and CoD performances were moderately correlated (25–35% of shared variance) in elite players [13,14]. Furthermore, Yanci et al. [7] showed that the correlation between jumping and CoD varied depending on the characteristics of test types. Similarly, Sekulic et al. [12] reported moderate to large correlations (0.40–0.58) between CoD and balance performances in male soccer players.

It is unknown why the above discrepancies between studies exist though one plausible, yet unresearched, factor could be the different physiological profiles associated with players’ playing positions [15]. For instance, strikers and full backs spend 20–40% more time sprinting than do midfielders or defenders [1,5,16]. Despite this, just one study, in recreational soccer players, conducted by Goral et al. [8], revealed that CoD performance was significantly worse in the midfielders compared with the goalkeepers (*p* < 0.05) and strikers (*p* < 0.05). Based upon the agility *t*-test results, midfielders were found to be significantly faster than goalkeepers (*p* < 0.05), whilst no differences were identified between players in other positions (*p* > 0.05). Thus far, several studies have examined the CoD performances of elite soccer players. However, the lack of studies that have evaluated CoD characteristics of soccer players, according to their on-field position, must be addressed. To optimise soccer-specific performance testing and training, research should be conducted to evaluate the relationship between CoD performance determinants, such as linear sprint speed and jump performances, with respect to a player’s position [1,5,15]. In light of the above, the main aim of this study was to determine the associations between measures of CoD, with and without the ball, and linear sprint speed, jump performance, and dynamic balance, with respect to playing position, in elite male soccer players. With regard to the relevant literature, we hypothesized that there would be significant differences in these factors across playing positions, potentially due to the varied demands imposed within position [8,15].

## 2. Material and Methods

### 2.1. Participants

Forty elite male soccer players (26.5 ± 2.1 yr), from two teams participating in the National Tunisian First League (The National Tunisian Soccer Team was positioned “14th” in the FIFA ranking, and “1st” in the CAF ranking in 2018, the time when the study was carried out), were enrolled in this study. All players were involved in systematic soccer training for at least 10 years. The study was carried out during the in-season period, with the players training 5–6 times per week, each session lasting between 60 and 90 min (min). Players were divided into three groups (Defenders (N = 14), Midfielders (N = 12) and Strikers (N = 14)) according to their game position. Participants’ characteristic is presented in Table 1. All were healthy, without any history of major lower limb injuries, and were not taking any medication at the time of the study. Written informed consent was obtained from every participant (or their parents for underage players) after receiving a verbal explanation of the potential risks and benefits resulting from study participation. The study was approved by the Ethical Committee on Human Research of the University of la Manouba, Tunisia (ethic code: Tn, UM2018-103), and was carried out in accordance with the Declaration of Helsinki.

### 2.2. Measures

All procedures were carried out during the second half of the competitive season. Before the commencement of the study, and prior to the initiation of testing, all players completed a one-week familiarization period (two sessions/week) with all testing procedures. During this time, players received standardised instructions on proper movement technique for each test, delivered by a certified strength and conditioning specialist. 

During the first session, each player’s height and body mass were collected using a wall-mounted stadiometer and electronic scale, respectively. The leg length (LL) was measured from the most distal end of the anterior superior iliac spine to the most distal end of the medial malleolus of each limb [10] and the body-mass-index (BMI) was calculated as mass per height squared (kg/m^2^). Skinfold thickness was measured to the nearest mm, except for low values (usually 5 mm or less) when it was taken to the nearest 0.5 mm [17]. These readings were made at four sites on all subjects, at the biceps, triceps, subscapular and supra-iliac areas. The sum of skinfolds measures was monitored with a Harpenden skinfold calipers (Baty International, West Sussex, England). After the anthropometric measurements were completed, players performed a 10-min standardized warm-up consisting of jogging, followed by a series of dynamic lower limbs exercises (e.g., lunges and skipping). On three non-consecutive test days, players performed two trials of the CoD test (with and without ball), horizontal jump test (Five Jump test), speed tests (5-m, and 20-m sprint time), and dynamic balance test (Y-balance test). The order of these tests was randomized. Within one week, all tests were repeatedly performed and intra-class correlation coefficients (ICC), together with standard error measurement (SEM), were assessed.

### 2.3. Change-of-Direction Tests

**Figure 1:**
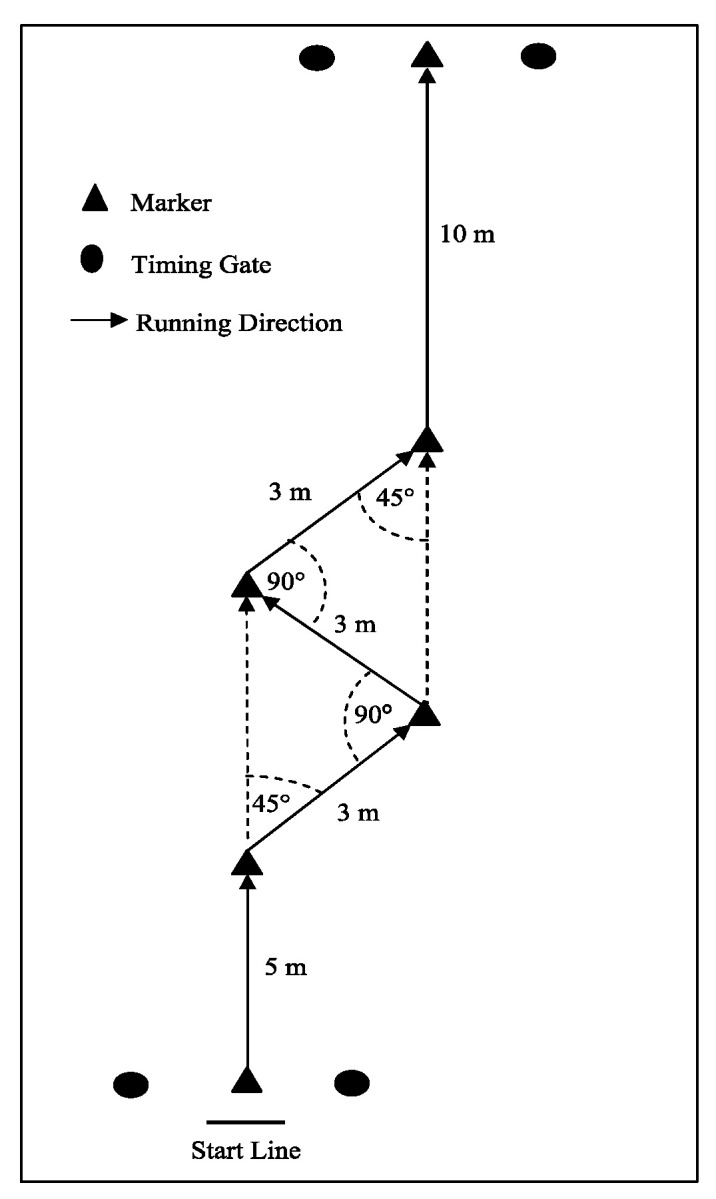
Change-of-direction and acceleration test (CODAT) with (CODATb) and without the ball (CODATwb) dimensions and completions route, m = meters.

Players performed change-of-direction and acceleration test (CODAT) with (CODATb), and without the ball (CODATwb) [18]. The dimensions and movement direction for the CODAT is shown in Figure 1. The CODAT was used for this assessment as it contains movement patterns common to many team sports (i.e., sprinting forwards while completing lateral cuts), and has been shown to be a valid and reliable assessment of change-of-direction speed [18]. Two timing gates were used, also positioned at a 1.2-m height and 2-m width; one positioned at the start, and the other at the finish of the test. Subjects started 30 cm behind the start line, were required to face forwards at all times during the CODAT, and stayed outside the markers when running. If subjects cut across or over a marker, the trial was stopped and another attempted. Three trials were completed with 3 min of recovery between trials. 

Performance time was recorded to the nearest 0.1 s, and the best time was used for further analyses [19,20].

### 2.4. Linear Speed

Linear sprint tests included the 5-m and 20-m sprint tests. For these tests, players were required to start with the front foot 5 cm behind the first timing gate. Photocells were positioned at the start line (0-m), and at 5-m and 20-m (finish-line) points, 0.4 m above the ground. Measurements were conducted with an accuracy of 0.01 (s). Tests were performed twice and were separated by a passive recovery of at least 5 min. The best performance speed was recorded and used for further analyses [11].

### 2.5. Horizontal Jump

The five-jump test (5JT) was used to assess horizontal jump in elite soccer players [21]. This test was performed on the grass with the players equipped with appropriate soccer boots. The 5JT consists of 5 consecutive strides with joined feet position at the start and end of the jumps. From the starting joined feet position, the participant was not allowed to perform any back step with any foot; rather, he had to directly jump to the front with a leg of his choice. After the first 4 strides, i.e., alternating left and right feet for 2 times each, he had to perform the last stride and end the test again with joined feet. If the player fell back on completion of the last stride, the test was performed again (only 2 cases of this happening in the present study). The 5JT performance was measured in centimeters with a tape measure from the front edge of the player’s feet at the starting position to the rear edge of the feet at the final position. The person assessing the landing had to focus on the last stride of the player in order to exactly determine the last foot print on the grass, as the players could not always stay on their feet on landing. The starting position was set on a fixed point [21].

### 2.6. Dynamic Balance

Dynamic balance was tested using the lower quarter Y balance test (YBT), as previously described by Fusco et al. [22] and Hammami et al. [10]. For the Y-balance test, participants placed their hands on their hips and began in a unilateral stance with their big toe behind the line on the center of the tape. Distances were then recorded by pushing a moveable floor target with the big toe in the three different directions, with trials performed with the dominant leg. Participants were required to keep the heel of the non-reaching leg on the platform, maintaining the balance in a single leg stance, and returning the reaching foot back to the starting point before attempting the next direction. Maximal reaching distances were recorded to the nearest 0.5 cm marker on the Y-balance kit. 

Balance performance was calculated as the Y-balance test score, obtained by dividing the sum of the maximal reached distances in each of the three directions, by three times the length of the lower limb (LL; measured from the most distal end of the anterior superior iliac spine to the most distal end of the medial malleolus of each limb). Thus, normalized values can be viewed as a percentage of the excursion distance in relation to the participant’s leg length [20]. Following the completion of the test trials, each participant was given a 1-min rest period and then conducted two test trials in each direction. The examiner manually measured the distance from the center of the grid to the touch point and the results were documented after each reach. A composite score (CS) was calculated and taken as the dependent variable using the following formula: CS = ([maximum anterior reach distance + maximum posteromedial reach distance + maximum posterolateral reach distance] / [leg length × 3]) × 100.

### 2.7. Statistical Analyses

Data are presented as group mean values and standard deviations (SD). Normal distribution of data was assessed and confirmed using the Shapiro–Wilk test. Between-group (i.e., defenders, midfielders and strikers) differences in measures of CODAT, sprint, balance and horizontal jump were computed using MANOVA. Test–retest reliability of the variables was computed using Cronbach’s model of ICCs and SEMs in accordance with the method introduced by Hopkins (2005) [23]. The strength of associations is reported by their correlation coefficient (r-value), level of significance (*p*-value), and the amount of variance explained (R^2^-value). R-Values of 0.10, 0.30 and 0.50 represent small, medium and large correlations, respectively [24]. Using the Fisher r-to-z transformation, average z-transformed correlation coefficients (rz-values) were calculated. 

These rz-values were used to compute differences relative to players’ playing positions [25]. The corresponding formula is: z = [*z*1…*z*2)/(1/(*n*1…3) + 1/(*n*2…3)]. Further, multiple linear stepwise multiple regression models were calculated to determine the most robust predictors of CODAT. Coefficients of determination (R^2^ × 100) were used to interpret the meaningfulness of the relationships [24]. Adjusted R-Squared and Variation Inflation Factors (VIF) were also calculated to explore potential multi-collinearity [26]. All data were subjected to a principal component analysis using “one” as the prior communality estimate [27]. Varimax orthogonal transformation was used to convert the set of physical and anthropometric variables into a set of linearly uncorrelated variables, termed principal components. Subsequently, the Keyer–Meyer–Olkin (KMO) measure of sampling adequacy (Kaiser) and variable uniqueness were computed to indicate independent contribution [28]. We conducted a posteriori power calculation to discern the achieved power in the present study. Indeed, based on the study sample size, number of variables entered into the multiple linear regression, and the effect sizes achieved, a power of 0.88 was found. The significance level was set at alpha 0.05. All analyses were performed using Statistical Package for Social Sciences (SPSS) version 23.0 (SPSS Inc., Chicago, IL., USA) and the JASP statistical package (JASP Team, 2018, jasp-stats.org, Amsterdam, The Netherlands).

## 3. Results

### 3.1. Reliability Analyses

Table 2 displays the test–retest reliability analyses for all the tests conducted. Cronbach’s alpha intra-class coefficient correlation showed good reliability for all tests. ICC-values ranged from 0.88 to 0.95, with a standard error of measurement (SEM) from 0.01 to 0.49 and a coefficient of variation of <5%. Paired *t*-tests showed no significant differences between the scores recorded during the two trials for all measured variables.

### 3.2. Between-Group Differences in Measures of Change of Direction, Linear Speed, Dynamic Balance and Jump Performance

The MANOVA analysis revealed no statistically significant between-group differences for all measures of CODAT, linear 5-m sprint, dynamic balance, and jump performance. However, a significant effect of group was found (F = 5.26, *p* < 0.02) on 20-m sprint. Forwards are faster than midfielders (*p* < 0.03; ES = 1.90) (Table 3).

### 3.3. Correlations between Measures of Change of Direction with Jump Performance, Speed and Balance

Analyses of the whole sample revealed significant positive correlations between CODATwb and sprint time (*p* < 0.001 and *p* < 0.03, for 5-m and 20-m sprint, respectively). Conversely, large negative correlations were observed between CODATwb and 5JT (*p* < 0.01) and YBT (*p* < 0.01). Furthermore, significant positive correlations were observed between CODATb and sprint times (large and moderate; *p* < 0.01 and *p* < 0.05, for 5-m and 20-m sprint, respectively). A large negative correlation was found between CODATb and 5JT (*p* < 0.01) and YBT (*p* < 0.001) (Table 4).

For the defender group, large positive correlations were observed between CODATwb and 5-m (*p* < 0.01) and 20-m (*p* < 0.001) sprint times. A large negative correlation of CODATwb with 5JT (*p* < 0.02) and YBT (*p* < 0.01) was also observed. A large positive correlation was observed between CODATb with 5-m sprint (*p* < 0.01) and a large negative correlation with 5JT (*p* < 0.01) and YBT (*p* < 0.01) (Table 4).

For the striker group, a large positive correlation was observed between CODATwb and 5-m sprint time (*p* < 0.01). Conversely, a large negative correlation was identified between CODATwb and the 5JT test (*p* < 0.01). Significant large negative correlations were observed between CODATb and 5JT and YBT (*p* < 0.01 and *p* < 0.01, respectively) (Table 4).

Finally, for the midfielder group, large positive correlations were observed between CODATwb and sprint time (*p* < 0.05 and *p* < 0.01, for 5-m and 20-m sprints, respectively) and large negative correlations with 5JT and YBT (*p* < 0.05 and *p* < 0.02, respectively). Significant large positive correlations were identified between CODATb and sprint times (*p* < 0.01 and *p* < 0.02, for 5-m and 20-m sprint, respectively). Conversely, large negative correlations were observed between CODATb and 5JT (*p* < 0.03) and YBT (*p* < 0.01) (Table 4).

### 3.4. Regression Analyses of Change-of-Direction and Linear Sprint, Dynamic Balance, and Jump Performance

Variation inflation factors (VIF) were calculated for all variables, where no variable had a VIF > 5 [26], suggesting that multicollinearity is not markedly influencing the results (CODATwb—2.5, CODATb—4.1, sprint 5-m—4.2, sprint 20-m—2.4, 5JT—2.5, CS-YBT—3.4). In addition, PCA suggested that all variables contributed independently, with Keyer–Meyer–Olkin (KMO) sampling adequacy indicating adequate sampling [28].

When examining the entire sample, the single best predictor of CODATwb was performance in the 5-m test, with an explained variance of 52% (*p* < 0.001). For CODATb, the Y-balance performance explained 68% of the variance of performance (*p* < 0.001) (Table 5).

## 4. Discussion

We believe this study is the first to examine the relationship between a CODAT test, with and without the ball, and proxies of speed, jump performance, and dynamic balance in elite soccer players. Overall, moderate to large correlations were observed between CODAT tests and linear sprint times, jump performance and dynamic balance. Additionally, there were differences in these associations dependent on players’ on-field positions. 

As expected, the results of this study demonstrate that the balance, jump performance, speed and CoD relationship in elite soccer players differ according to the specific demands of soccer play. In this way, short sprint speed is an important determinant of a player’s ability to change direction whether they are in possession of the ball or without the ball. On this basis, short sprint speed should be a priority for coaches to address as it is likely to be important across all situational variables within a game of soccer.

Previously, several CoD, jumping, balance and speed tests in elite soccer players were proposed as necessary assessments for that sport [8,11,15,21]. However, few studies have examined the possible relationships between these outcome measures with regard to players’ positions. Our results support the findings previously reported by Kapidzic et al. [6], who found a significant correlation between the 10-m acceleration test and the zig-zag preplanned CoD test results (r = 0.34; *p* = 0.01). The strong influence of sprinting performance on CoD scores is a logical consequence of the nature of this test, as it is considered a preplanned agility test with a moment of zero velocity occurring throughout test execution. 

Although different combinations of plyometric, strength and complex training could assist in the development of this capacity for elite soccer players [29], the focus of neuromuscular training should be towards improving CoD by simultaneously developing jump performance, acceleration, and maximal sprinting speed capacities [11]. In this way, a coach can adopt several complementary training modalities, such as short sprint and plyometric training, which can independently contribute to the development of CoD in soccer players. Future studies should focus on collecting longitudinal data to confirm the current observations regarding the development of CoD within elite soccer players. 

The associations observed between horizontal jumping, balance test performance and CoD ability suggest the influence of neuromuscular factors involved in rapid CoD, in addition to some cognitive abilities [11]. In addition, the biomechanical and physiological similarities between jumping and CoD, which are observed throughout each “stop-and-go” movement during these actions, could be the reason behind the positive associations observed between jumping abilities and CoD scores. With these points taken together, a player must possess the physical attributes to efficiently negotiate the CoD demands of soccer, but also the cognitive capability to perceive and react within a very short timeframe during play. Thus, the influence of these factors on pre-planned CoD performance development and the use of further dynamic tests (on stabliometric platforms and using unstable surfaces) that mimic the nature of dynamic movement, should be considered in future studies with tests to determine true agility performance yet to emerge in the literature. 

Concerning players’ specific field positions, some relevant and novel relationship were also found. Positional challenges are determined by the varying physical demands imposed on players during soccer matches in the different areas of the field. For instance, although full backs require high speed and muscular power to pass and evade opponents along a relatively straight course, midfielders must be able to accelerate with changes of direction on a more frequent basis [15]. Information such as this could therefore be useful for the identification of game-specific strengths and weakness of players with regard to their specific playing position, thus facilitating a more focused training regime. On this, Brahim et al. [30] showed that midfielders tend to have the best CoD ability. According to Boone et al. [15], strikers are significantly faster than players in all other positions. This was reinforced by Gil et al. [31], who found that strikers were the fastest players on the team with goalkeepers being the slowest. Davies [32] stated that players spend most of a game without possession, underlining the importance of addressing speed and agility both with, and without, the ball. Coaches must consider these differences in formulating training programs for players and our results further contribute to the knowledge on the highly variable challenges associated with the different playing positions in soccer.

One novel aspect of our findings was the significant association observed between balance and CoD test performances observed for all players. Thus, from these results, it may be suggested that the motor control required for optimal CoD in soccer is partially dependent on dynamic balance capabilities. Balance has rarely been studied in relation to CoD ability, although some researchers have previously noted its importance in enhancing CoD [10], whilst others have recognised it as a characteristic of efficient CoD [29]. The influence of balance on CoD could relate to one’s ability to accurately coordinate the timing and action of skeletal muscles [33] thus maintaining appropriate postural stability and, therefore, the ability to maintain balance [22]. This hypothesis could be further verified through analysis of the patterns of motion, along with the mechanical demands, of the shuttle run test that was performed. For the successful execution of this test, the ability to perform rapid accelerations and decelerations, and to quickly change position from side to side, as in soccer play, is crucial. Given that these actions cause frequent perturbations of the center of gravity, which requires efficient neuromuscular control, it is reasonable to assume that players’ ability to efficiently maintain dynamic balance may positively affect the high-speed athletic maneuvers required in soccer. It is interesting that CoDwb shared a weaker relationship with YBT in strikers and though it is unclear as to why this result occurred, coaches could consider this finding when formulating training programs for strikers, defenders and midfielders alike. 

Accordingly, exercises to improve CoD, and to develop dynamic balance, must be given due consideration by coaches [34] with potentially less emphasis placed on this in attacking players who are in possession of the ball. Moreover, further studies are required to appropriately evaluate the additive advantage of combining balance training with more traditional forms of speed and power training. Similarly, researchers should also consider the potential of alternative characteristics, such as anthropometrics and muscle strength, to impact upon YBT performances [22].

This study is not without limitations; the sample size of each positional group was small. Therefore, this study is preliminary. However, it is difficult and almost impossible to recruit large sample sizes in elite sport, especially in a highly professionalized elite sport such as soccer. While our results provide interesting information for coaches and strength and conditioning specialists, they have to be interpreted with caution and should be verified in future studies. Another limitation related to the fact that only two teams from the same league were included; therefore, it is unknown if different results would be observed when including elite players from other countries and competitive levels.

## 5. Conclusions

In conclusion, moderate to large correlations between CoD, dynamic balance, jump performance and linear sprint times in elite soccer players, according to players’ positions, were found. This suggests that there could be interdependent positive transfer effects, from training that is singularly focused on dynamic balance, sprint speed and muscle power, to CoD performance. Despite this, the present findings are based on cross-sectional data which does not conclusively allow for cause-and-effect relationships to be determined. Nonetheless, the findings suggest that when designing training programmes aimed to improve CoD in soccer players, coaches should pay attention to these specific associations and players’ on-field positions, which can necessitate an individualized approach to programme design.

## Figures and Tables

**Table 1:** Participants’ anthropometric characteristics by player’s positions.

	Midfielders (*n* = 12)	Defenders (*n* = 14)	Strikers (*n* = 14)	Total (*n* = 40)	F	Sig. *p*-Value
**Age (yr)**	25.28 ± 6.17	31.83 ± 19.15	25.80 ± 3.23	27.76 ± 11.77	0.62	0.55
**Height (cm)**	181.50 ± 6.44	170.00 ± 39.21	178.57 ± 5.09	176.45 ± 23.02	0.42	0.66
**SH (cm)**	89.50 ± 6.57	89.08 ± 13.08	89.00 ± 4.47	89.18 ± 8.47	0.00	0.99
**LL (cm)**	92.00 ± 7.87	85.42 ± 14.80	89.57 ± 4.43	88.85 ± 9.97	0.71	0.51
**BM (kg)**	77.60 ± 6.16	76.91 ± 18.71	75.07 ± 5.70	76.47 ± 11.49	0.08	0.93
**BMI (kg/m^2^)**	23.56 ± 1.50	30.11 ± 18.86	23.51 ± 1.04	25.83 ± 11.12	0.78	0.48
**BF (%)**	11.38 ± 2.83	12.16 ± 3.40	10.97 ± 2.34	11.51 ± 2.79	0.30	0.74

Notes: Values are means and standard deviations (SD), SH = Sitting Height; LL = Leg Length; BM = body mass; BF = body fat; BMI = body mass index.

**Table 2:** Test–retest reliability of the applied change-of-direction, balance, jump, and speed tests.

Criterion Measures	ICC_3.1_ (95% CI)	SEM	CV (%)
**CODATwb (s)**	0.92 (0.75–0.97)	0.04	2.75
**CODATb (s)**	0.88 (0.71–0.95)	0.06	2.86
**Sprint 5-m (s)**	0.94 (0.85–0.97)	0.01	2.08
**Sprint 20-m (s)**	0.95 (0.88–0.98)	0.01	1.14
**5JT (m)**	0.91 (0.77–0.97)	0.10	2.44
**CS-YBT (%)**	0.91 (0.78–0.96)	0.49	2.40

Notes. ICC = intraclass correlation coefficient; SEM = standard error of measurements; CV (%) = coefficient of variation percent; CODATwb = change-of-direction and acceleration test without a ball; CODATb = change-of-direction and acceleration test with a ball; 5JT = five jump test; CS-YBT = composite score during the y-balance test.

**Table 3:** Performance variables for change-of-direction, balance, speed, and jump measurements according to player’s positions.

	Midfielders (*n* = 12)	Defenders (*n* = 14)	Strikers (*n* = 14)	Total (*n* = 40)	F	Sig. *p*-Value
**CODATwb (s)**	5.28 ± 0.28	5.13 ± 0.18	5.09 ± 0.40	5.16 ± 0.29	0.71	0.50
**CODATb (s)**	6.73 ± 0.63	6.75 ± 0.24	6.27 ± 0.35	6.57 ± 0.46	2.78	0.09
**Sprint 5 (m)**	0.87 ± 0.03	0.84 ± 0.06	0.81±0.04	0.84 ± 0.05	2.69	0.09
**Sprint 20 (m)**	2.84 ± 0.09	2.75 ± 0.10	2.67 ± 0.08	2.75 ± 0.11	5.26	0.02
**5JT (m)**	12.46 ± 1.07	12.85 ± 0.86	12.96 ± 0.57	12.77 ± 0.83	0.61	0.56
**CS-YBT (%)**	92.03 ± 6.90	95.01 ± 5.89	101.65 ± 7.72	96.44 ± 7.70	3.39	0.06

**Notes**: Values are means and standard deviations (SD); CODATwb = change-of-direction and acceleration test without a ball; CODATb = change-of-direction and acceleration test with a ball; 5JT = five jump test; CS-YBT = composite score during the y-balance test.

**Table 4:** Correlations between measures of change-of-direction and speed, jump and balance assessed in a sample of 40 elite soccer players according to players’ positions. Data represent the final step in each stepwise model.

WHOLE SAMPLE (*n* = 40)										
Variables	Sprint (s)				Jump		Dynamic Balance			
	5 (m)		20 (m)		5 JT (m)		CS YBT (%)			
	r-Value (r^2^)	r_z_-Value	r-Value (r^2^)	r_z_-Value	r-Value (r^2^)	r_z_-Value	r-Value (r^2^)	r_z_-Value	Mean r_z_-Value	Mean r_z_-Value (r^2^)
CODATwb	0.72 ** (0.52)	0.91	0.54 * (0.29)	0.60	−0.62 ** (0.38)	0.73	−0.50 ** (0.25)	0.55	0.698	0.595 (35)
CODATb	0.52 ** (0.27)	0.58	0.45 * (0.21)	20 (m)	−0.64 ** (0.42)	0.76	−0.83 ** (0.68)		0.753	0.610 (37)
**Mean rz-value †**	N/A	0.74	N/A	0.54	N/A	0.74	N/A	0.87	0.723	0.605
**Mean r-value (r^2^) †**	N/A	0.62 (38)	N/A	0.50 (25)	N/A	0.63 (40)	N/A	0.67 (44)	-	-
**DEFENDERS (*n* = 14)**										
**Variables**	**Sprint (s)**				**Jump**		**Dynamic Balance**			
	**5 (m)**		**20 (m)**		**5 JT (m)**		**CS YBT (** **%)**			
	**r-Value (r^2^)**	**r_z_-Value**	**r-Value (r^2^)**	**r_z_-Value**	**r-Value (r^2^)**	**r_z_-Value**	**r-Value (r^2^)**	**r_z_-Value**	**Mean r_z_-Value**	**Mean r_z_-Value (r^2^)**
CODATwb	0.83 ** (0.69)	1.19	0.96 ** (0.92)	1.95	−0.57 * (0.32)	0.65	−0.83 ** (0.68)	1.19	1.245	0.798 (64)
CODATb	0.78 ** (0.60)	1.05	0.40 (0.16)	0.42	−0.76 ** (0.58)	0.99	−0.85 ** (0.73)	1.26	0.930	0.698 (49)
**Mean rz-value †**	N/A	1.12	N/A	1.18	N/A	0.82	N/A	1.22	1.085	0.775 (60)
**Mean r-value (r^2^) †**	N/A	0.81 (65)	N/A	0.68 (46)	N/A	0.77 (44)	N/A	0.84 (71)	-	-
**MIDFIELDERS (*n* = 12)**										
**Variables**	**Sprint (s)**				**Jump**		**Dynamic Balance**			
	**5 (m)**		**20 (m)**		**5 JT (m)**		**CS YBT** **%**			
	**r-Value (r^2^)**	**r_z_-Value**	**r-Value (r^2^)**	**r_z_-Value**	**r-Value (r^2^)**	**r_z_-Value**	**r-Value (r^2^)**	**r_z_-Value**	**Mean r_z_-Value**	**Mean r_z_-Value (r^2^)**
CODATwb	0.58 * (0.34)	0.66	0.82 ** (0.68)	1.16	−0.58 * (0.34)	0.66	−0.62 * (0.39)	0.73	0.803	0.650 (42)
CODATb	0.68 * (0.46)	0.83	0.73 * (0.53)	0.93	−0.67 * (0.44)	0.81	−0.89 * (0.79)	1.42	0.998	0.743 (55)
**Mean rz-value †**	N/A	0.74	N/A	1.04	N/A	0.73	N/A	1.07	0.895	0.708 (50)
**Mean r-value (r^2^) †**	N/A	0.63 (40)	N/A	0.78 (60)	N/A	0.66 (43)	N/A	0.76 (57)	-	-
**STRIKERS (*n* = 14)**										
**Variables**	**Sprint (s)**				**Jump**		**Dynamic Balance**			
	**5 (m)**		**20 (m)**		**5 JT (m)**		**CS YBT** **%**			
	**r-Value**	**r_z_-Value**	**r-Value (r^2^)**	**r_z_-Value**	**r-Value (r^2^)**	**r_z_-Value**	**r-Value (r^2^)**	**r_z_-Value**	**Mean r_z_-Value**	**Mean r_z_-Value (r^2^)**
CODATwb	0.86 ** (0.75)	1.29	0.14 (0.02)	0.14	−0.84 ** (0.71)	1.22	−0.27 (0.007)	0.28	0.733	0.528 (28)
CODATb	0.12 (0.01)	0.12	0.44 (0.19)	0.47	−0.75 ** (0.56)	0.78	−0.76 ** (0.58)	1.00	0.593	0.518 (27)
**Mean rz-value †**	N/A	0.70	N/A	0.30	N/A	1	N/A	0.64	0.660	0.525 (28)
**Mean r-value (r^2^) †**	N/A	0.49 (24)	N/A	0.29 (8)	N/A	0.80 (63)	N/A	0.52 (27)	-	-

Notes: N/A = not applicable; * *p* < 0.05; ** *p* < 0.01; r^2^ = coefficient of determination which was calculated by squaring the r-value and by multiplying it by 100 to obtain explained variance (%); † mean r_z_-values were transformed in r-values to obtain r^2^; CODATwb = change of direction and acceleration test without a ball; CODATb = change-of-direction and acceleration test with a ball; 5JT = five jump test; CS-YBT = composite score during the y-balance test.

**Table 5:** Stepwise linear regression analyses with measures of change-of-direction (CODATwb or CODATb), as criterion variable and speed (Sprint 5-m, Sprint 20-m), balance (CS-YBT) and jump (5JT), as predictor variables in a sample of 40 elite soccer players. Data represent the final step in each stepwise model.

	Model		Unstandardized Coefficients		Coefficients	t	Sig.	R Square (R^2^) (Adjusted R^2^)
			B	Std. Error	Beta			
**CODATwb**	1	(Constant)	1.50	0.83		1.81	0.08	
		Sprint 5-m	4.36	0.99	0.72	4.42	0.000	0.52 (0.50)
**CODATb**	1	(Constant)	11.38	0.77		14.73	0.000	
		CS-YBT	−0.05	0.01	−0.83	−6.25	0.000	0.68 (0.66)

Notes: CODATwb = change-of-direction and acceleration test without a ball; CODATb = change-of-direction and acceleration test with a ball; CS-YBT = composite score during the y-balance test.
